# Inadequate intake of energy and nutrients: A comparative cross‐sectional study between sport and nonsport science university students of southern Ethiopia

**DOI:** 10.1002/fsn3.3779

**Published:** 2023-11-02

**Authors:** Kebede Awgechew, Beruk Berhanu Desalegn, Addisalem Mesfin

**Affiliations:** ^1^ School of Human Nutrition, Food Science and Technology, College of Agriculture Hawassa University Hawassa Ethiopia

**Keywords:** dietary intake, Ethiopia, sport science, university students

## Abstract

This study aimed to investigate and compare the energy and selected nutrient intakes of sport science and nonsport science university students of Southern Ethiopia. Multiple‐day dietary data were collected from 166 university students (76 sport science and 90 nonsport sciences). Average daily energy and nutrient intake, and inadequate intakes were calculated using NutriSurvey (NS). There were significant differences (*p* < .05) in the median intakes of energy, total carbohydrate, and vitamin B_1_ between female students from the sport science and nonsport science groups, but only the median intake of iron was significantly different (*p* < .05) between the male sport and nonsport science student groups. The prevalence of inadequate intake of vitamin B_1_ was significantly (*p* < .05) higher in the male and female from the nonsport science groups compared to the male and female student groups in the sport science, respectively, whereas the prevalence of inadequate iron intake by the male sport science students' group was significantly (*p* < .05) higher compared to their counterparts. Similarly, the prevalence of inadequate energy among the females from the sport science group was significantly (*p* < .05) higher compared to the female students from the nonsport science department group. The prevalence of inadequate intake of dietary energy and the majority of the nutrients (protein, fat, vitamin A, B_1_, B_2_, and magnesium) were high (>50%) in selected university students. The energy and majority of nutrient intakes by the students in the selected universities of southern Ethiopia were suboptimal. Therefore, activities that will improve the dietary intake of university students should include weekly meal plan revision considering their average recommended nutrient intake (RNI).

## Abstract 2

This study aimed to investigate and compare the energy and selected nutrient intakes of sport science and nonsport science university students of Southern Ethiopia. The energy and majority of nutrient intakes by the students in the selected universities of southern Ethiopia were suboptimal. Therefore, activities that will improve the dietary intake of university students should include weekly meal plan revision considering their average recommended nutrient intake (RNI).

## 1 BACKGROUND

Proper dietary practice is important to human health (Bhandari et al., 2016). Improper and inadequate dietary intake affects individuals' nutritional status, leading to different undernutrition problems, including micronutrient deficiency. These problems are high in developing countries, which affect the physical, mental, social, and economic status of individuals, as a result, burdening these nations. Besides, less attention has been given to adolescents (10–19 years old) and young adults (20–24 years old) health and nutrition are living in low‐ and middle‐income countries (LMICs) covering about 90% of the total adolescents' population globally (Das et al., 2017). According to the joint FAO/WHO/UNU Expert Consultation group, physical activity level (PAL) is classified into three: sedentary or light activity lifestyle, active or moderately active lifestyle, and vigorous or vigorously active lifestyle, which has a direct relationship with the daily energy expenditure and estimated energy need. For instance, a 50‐kg male aged between 18 and 29.9 years with low PAL (1.45) requires less estimated energy requirement compared to an individual with the same sex and age and sex individual, but with higher PAL (2.2) (2100 vs. 3200 kcal, respectively). This indicates that those individuals such as athletes are expected to expend more energy; as a result, more energy is needed from energy source macronutrients as per recommendations, otherwise, the health of specific individuals will be affected (FAO/WHO/UNU, 2001).

In Ethiopia, most of the students join university education starting from the age of late adolescence up to early young adulthood (18–20 years), in which pubertal maturation ends and adoption of adult roles and responsibilities is initiated, but this maturation might sometimes be extended in poor countries, thus their growth may not be complete in the expected age (Das et al., 2017; Patton et al., 2016). Therefore, proper nutrition between 18 and 20 years is vital and contributes to the health and well‐being of individuals, which benefits today, for the coming decades, and for the next generation of a given country (Patton et al., 2016).

According to the Federal Democratic Republic of Ethiopia‐Ministry of Education (FDRE‐MOE), about 147,640 first‐year students were expected to join 47 government universities in 2020/2021. While these students were attending their high school education, most of them were expected to live together with their families and share family foods and diversified dietary practices. Coupled with a new independent life, living in the same boarding hostels and joining a cafeteria at universities, and transitioning from high school to university‐level education, politics, religion, campus norms, culture, and economic problems, students might suffer from physical and psychological stresses (Dyson & Renk, 2006; Kabir et al., 2018). These may also affect their eating behaviors and food habits, as they are usually influenced by different factors such as individual, social, and environmental factors in a given context (Deliens et al., 2014; Kabir et al., 2018). A study in Belgium confirmed that the transitional period from secondary level to graduate level is critical for students and these students were often engaged in unhealthy dietary habits and had poor nutritional intake (Deliens et al., 2014). Furthermore, studies in different countries also revealed that proper dieting of university/college students was positively associated with their academic performance (Burrows et al., 2017; Deliens et al., 2014; Ul Haq et al., 2018).

In the last 10 years, there has been a quick expansion of sport science departments throughout Ethiopian universities. However, unlike other students pursuing their first degrees in different disciplines, sport science department students require extra energy and nutrient intake due to the nature of the courses they are taking. This requires special attention for the sport science students considering their daily requirements. Yet, this is not happening in Ethiopian universities, as the quality and quantity of the food provided to the students depend mainly on the daily budget allotted for an individual student. Thus, the revolving meal plan or menu which is designed for each day of the week at each Ethiopian University depends on the food budget per individual student, with some exceptional festivities such as a holiday. Otherwise, to the best of the authors' knowledge, there is no special consideration practiced for Ethiopian students studying a sport science, demanding extra energy and nutrients, due to their routine extensive exercise engagement as part of their academic exercise and requirement. This may also be coupled with designing a sport science curriculum without considering the distribution of practical intensive courses along different academic semesters with student dietary intake, thus leading to the sport science students being more inefficient in their academic performance in general (Federal Democratic Republic of Ethiopia Ministry of Education (FDRE‐MoE), 2013). Despite these, to the authors' knowledge, no published study documented the dietary energy and nutrient intake for Ethiopian university students in general and more specifically for sport science students.

Therefore, this study aimed to investigate and compare the energy and selected nutrient intakes of sport science and nonsport science university students of Southern Ethiopia.

## 2 MATERIALS AND METHODS

### 2.1 Study setting and period

This study was conducted between December 15, 2017 and January 30, 2018 in three first‐generation universities (FGUs) located in the Southern part of Ethiopia, namely, Hawassa, Dilla, and Arba Minch. According to the Federal Ministry of Education of Ethiopia (FMOE), the universities under the FGUs category were established earlier, and have better facilities, equipment, and staff profiles compared to those universities under the category of second‐ and third‐generation universities.

### 2.2 Study design and population

This study followed an institution‐based comparative cross‐sectional study design. The study populations were the first‐year undergraduate regular sport science and nonsport science students who were registered in the 2017/2018 academic year in departments under the College of Natural and Computational Sciences (CNCS) in the selected three universities. The student service affairs directorates in Ethiopian universities provide cafeteria, boarding, medical, and recreational services mainly to regular undergraduate students based on the budget allocated by the Government of Ethiopia, which will be paid by the students later after they graduate from the study and are employed. In each university, more specifically at the colleges/campuses where this study was conducted, the food prepared and served in the cafeterias were prepared in central kitchens. In these cafeterias, there are daily meal menus for each of the 7 days in a week, but these may be slightly different from campus to campus, with preference of the meal prepared differently during fasting days or seasons. Furthermore, there could be a special menu that can be prepared for a few students during illness and food habits. Moreover, in these cafeterias, the raw materials that are needed for preparing the meal are estimated, which is dependent on the number of the student and their budget. However, some students who were not using the cafeteria service under the directorate during the study period received an equivalent budget allocated for the meal from the directorate in a monthly period. Therefore, they will purchase their food within and outside the campuses they are living using the money provided by the university and other sources. Those students who were obtaining cafeteria service (breakfast, lunch, and dinner) through the three universities' student service affairs directorate during the study period were eligible and included in the study. Of the eligible students, those who were ill/with a medical problem during the data collection period and those who had impairment/disability were excluded from this study.

### 2.3 Sample size and sampling techniques

During the study period, there were 1061, 319, and 903 total registered first‐year students under the CNCS in the 2017/2018 academic year at Hawassa, Dilla, and Arba Minch universities, respectively. Of them, the number of sport and nonsport science students registered in the same academic year at Hawassa, Dilla, and Arba Minch universities were 210 and 851; 49 and 270; and 120 and 783, respectively. The sample size was calculated by using a G‐power software version 3.0 with an assumption of the mean difference between two independent groups, for which a *t*‐test was used. For this, a medium effect size (.5), standard deviation difference between means (2‐tailed), with α error probability of 0.05 and powers (1‐β error probability) of 0.90 to allow comparison of two groups of one or more of the outcomes and allocation ratio between groups 1 and 2 was 1.1. The estimated sample size was 172 (82 for sport science and 90 for nonsport science student groups) (Table S1).

The study participants were selected from each university using a multistage stratified sampling technique. Initially, the list of universities located in southern Ethiopia was prepared and they were stratified into first‐, second‐, and third‐generation universities based on the FMOE category. Of which, the first‐generation stratum was purposively included, as universities in this category were believed to have well‐established and better cafeteria services. In this stratum, Hawassa, Dilla, and Arba Minch universities were grouped, and thus, all were included in this study. Due to sport science department being under the CNCS in the three universities included in this study, and the cafeteria services provided to the students in the CNCS were the same, CNCS was purposively selected. Following that, two strata, sport science, and nonsport science were created and a proportional allocation of the samples was done. Furthermore, the proportional allocation for both sexes was done, for each stratum, at each university. Finally, the list of students was prepared and a corresponding three‐digit number was assigned to each, and the sample was randomly selected from the coded list using an Excel sheet.

### 2.4 Data collection method

Data were collected by 10 trained and experienced technical assistants at Hawassa University. The principal investigator and one additional supervisor, who was also staff at Hawassa University, were also recruited. The sociodemographic information was collected using a structured and pretested questionnaire. The questionnaire was first prepared in English, which was translated into the Amharic language for interview by the principal investigator and checked by different professionals related to the study. Because Amharic is the national language, most of the students can speak, listen, write, and understand it, the questionnaire was translated to it. Furthermore, the 7 days of dietary information was collected with the food record method using a validated standard questionnaire in southern Ethiopia and translated into Amharic language (Alemayehu et al., 2011). In the questionnaire, the name of the food, preparation method, weight of food served and eaten, and weight of the leftovers were included. All the foods consumed and drinks served to the study students were directly weighed and recorded before and after cooking with a manual weight scale. For instance, the raw materials that were used for preparing for given students were listed first, followed by the weighing recorded on the dispatch record sheet at the cafeteria store. Because there was no standard recipe to be used for food preparations, whenever the cooks felt that the quality of the prepared food was not good, they might ask for additional ingredients so that they might try to maintain the quality to be consistent at some level. Whenever additional raw materials were used, the added raw materials were weighted and summed in the weight of the previous raw materials used. On the contrary, whenever the cooks felt that the raw taken were excess, the excesses were subtracted from the total raw materials required, which helped to estimate the total dietary energy and nutrient intake calculated much closer to the actual intake. These variations mainly occur in the raw materials that were used for the preparation of the stews such as red pepper‐, peas‐, lentil‐, meat–potato, and cabbage‐based stews which were commonly prepared in the student cafeterias of the studied universities. To estimate the average weight of injera (a fermented, pancake‐like soft, circular flatbread similar to chapatti with small bubbly structures or eyes (honey‐comb‐like holes) on its top surface and mainly produced from teff, barley sorghum, maize, and wheat flours) and also bread, at least the weight of 10 pieces was measured and the average for the respective food was taken. The weighing of the food served to students during breakfast, lunch, and dinner each day for each student included in this study was taken for seven consecutive days. An electronic portable scale (CS200 Ohaus Corp., CHINA) was used to weigh the foods with a precision of ±1 g. The plate was weighed and the scale was adjusted to zero before the dish was served. Leftovers (a portion of the meal that is left and brought by the student to discard in the discard place/bin) were weighed and subtracted from the original weight of the food dish to obtain the actual weight of the food consumed by each student. Whenever appropriate, separate weighing of the sauce/injera/bread was done for the leftovers from an individual student food provided if it was easy to do so; otherwise, when it was believed to be mixed thoroughly while eating, to estimate the proportion of the meal eaten by the individual student, the leftover was estimated proportionally from the origin food provided to the student and deducted from the whole portion weight of the meal provided for an individual student. However, to identify the whole portion weight of the meal and also the specific ingredients provided for an individual student, the injera/bread that was provided, and the specific sauce given with the ladle are first estimated. The procedures mentioned were followed in the seven data collection days. As the interest of this was to identify the actual food provided for the students in the cafeteria of the universities and also assuming those students included in this study were dependent exclusively on cafeteria food, due to their economic status, we intended to assess the usual intake from the cafeteria. In a few individual cases (*n* = 5), additional 1–2 tea/coffee were taken per day and the contribution of the specific beverages drunk by the specific student was excluded to capture the usual dietary intake of the students from the university cafeteria.

### 2.5 Estimation of dietary energy, nutrient intakes, and their inadequate intakes

A food composition database was adapted mainly from the Ethiopian food composition table. For the missing nutrient values for some food items (Ethiopian Health and Research Institute, 1998; Ethiopian Nutrition Institute, 1981), the food compositions analyzed elsewhere in Ethiopia were used (Abebe et al., 2007; Umeta et al., 2005), and the USDA food composition database (USDA, 2019) was adapted, considering the moisture content difference between the same food item. The prepared food composition database was entered into NutriSurvey (NS) software version 2007 program (German Bundes Leben Smittelschlüssel (BLS, version II.3, 1999)). The estimated amount of each food item consumed in the 7 days was also entered into the NS software, and thus the estimated average daily intake of energy and selected nutrients in this study was calculated for each individual. Finally, the output of the estimated average daily dietary energy and nutrient intakes for the whole participants was generated in Excel and used for further analysis in SPSS software version 20.0.

For identifying the adequate intakes for energy and nutrients included in this study, two‐thirds of the recommended nutrient intake (RNI) was used as a cut‐off point (Bosha et al., 2019; Jati et al., 2014; Megersa, 2020), for which age and sex were considered.

### 2.6 Data management and analysis

Data analyses were carried out using SPSS version 20. Comparison of frequency (%) between groups for the categorical data was done using the chi‐square test. For estimated energy and nutrient intake data, normality was checked using the Kolmogorov–Smirnov test separately for each group and all of them were found to be not normally distributed. Thus, the comparison of the energy and nutrient intake values between groups was done using the Mann–Whitney U test, and the results are presented as median and interquartile ranges (IQR). The median energy and nutrient intake of all students were compared with the reported by the FAO/WHO for the different age groups and sex (FAO/WHO, 2001; Institute of Medicine (IOM), 2010, 2011; Otten et al., 2006). The statistical significance level was declared at *p* ≤ .05.

## 3 RESULTS

### 3.1 Sociodemographic characteristics

The information of sociodemographic characteristics of the study participants is presented in Table 1. A total of 166 out of 172 university students participated in the study who fulfilled the inclusion and exclusion criteria. Therefore, the response rate of this study was 96.5%. A slightly higher proportion of male students participated, both from the sport science (59.2%) and nonsport science (52.2%) categories compared to their counter female students' group. The majority of the students in the sport science (86.8%) and nonsport science (85.6%) categories were aged between 19 and 21 years. The proportion of female students who participated from the sport science department was higher compared to female students from the nonsport science category.

**TABLE 1:** Sociodemographic and economic characteristics of sport science and nonsport science students in the first‐generation universities of southern Ethiopia, January 2018.

Variables	SSS		Total (*N* = 76)	NSSS			*χ* ^2^
	Male (*N* = 45)	Female (*N* = 31)		Male (*n* = 45)	Female (*n* = 31)	Total (*N* = 90)	
	*N* (%)	*N* (%)	*N* (%)	*N* (%)	*N* (%)	*N* (%)	*p*‐Value
Age category in years							.622
19–21	44 (97.8)	23 (74.2)	67 (86.8)	39 (83)	38 (88.4)	77 (85.6)	
22–24	1 (2.2)	8 (25.8)	10 (13.2)	8 (17)	5 (11.6)	13 (14.4)	
Median (IQR)	20 (19, 20)	20 (19, 22)	20 (19,20)	20 (19,21)	20 (19,20)	20 (19,21)	.058
Ethnicity							
Amhara	11 (24.4)	11 (35.5)	22 (28.9)	17 (36.2)	17 (39.5)	34 (37.8)	.084
Oromo	11 (24.4)	6 (19.4)	17 (22.4)	21 (44.7)	10 (23.3)	31 (34.4)	
Sidama	13 (28.9)	8 (25.8)	21 (27.6)	6 (12.8)	10 (23.3)	16 (17.8)	
Wolaita	5 (11.1)	5 (16.1)	10 (13.2)	2 (4.3)	4 (9.3)	6 (6.7)	
Others	5 (11.1)	1 (3.2)	6 (8.9)	1 (2.1)	2 (4.7)	3 (3.3)	
Religion							
Orthodox	13 (28.9)	14 (45.2)	27 (35.5)	21 (44.7)	15 (34.9)	38 (42.2)	.818
Muslim	5 (11.1)	4 (12.9)	9 (11.8)	5 (10.6)	5 (11.6)	8 (8.9)	
Protestant	20 (44.4)	10 (32.3)	30 (39.5)	17 (36.2)	19 (44.2)	36 (40)	
Catholic	7 (15.6)	3 (9.7)	10 (13.2)	4 (8.5)	4 (9.3)	8 (8.9)	
Place of residence							
Rural	16 (35.6)	18 (58.1)	34 (44.7)	20 (51.2)	22 (51.2)	42 (46.7)	.804
Urban	29 (64.4)	13 (41.9)	42 (55.3)	27 (57.4)	21 (48.8)	48 (53.3)	
Secondary and preparatory school attended							
Government school	42 (93.3)	29 (93.5)	71 (93.4)	43 (91.5)	39 (90.7)	82 (91.1)	.581
Private school	3 (6.7)	2 (6.5)	5 (6.6)	4 (8.5)	4 (9.3)	8 (8.9)	

*Note*: Categorical data are presented as a proportion and compared using a Chi‐square test (*χ* ^2^); continuous data are presented as median (IQR) and compared using a Mann–Whitney U‐test: statistical significance declared at *p* ≤ .05.

Abbreviations: NSSS, nonsport science students; SSS, sport science students.

Slightly more than half (53.3% and 55.3%) of the participants from the nonsport science and sport science groups were living in urban areas before they joined any of the three universities included in this study. About nine of the 10 university students who participated in this study attended their preparatory education at government schools. However, none of the sociodemographic variables included had shown a significant difference between the students from the sport and nonsport science group in both sexes (Table 1).

### 3.2 Median dietary energy and other selected nutrient intakes

The average daily estimated median (25th, 75th) intakes of energy and nutrients for the sport science and non‐sport science students over a 7‐day period are presented in Tables 2 and 3. The average median intake of iron for the male sport science students was significantly lower compared to the nonsport science male students, whereas the median energy and macro‐ and micronutrient intakes were not statistically different between the two groups (Table 2).

**TABLE 2:** Estimated daily median intakes (Q1, Q3) of energy and other selected nutrients of male sport science and nonsport science students in the first‐generation universities of Southern Ethiopia, Ethiopia, January 2018.

Variables	Male sport science and nonsport science (*N* = 92)				
	MSS (*n* = 45)		MNSS (*n* = 47)		*p*‐Value
	Median	Quartile (Q1, Q3)	Median	Quartile (Q1, Q3)	
Energy (kcal)	2035	1955, 2140	2036	1908, 2113	.444
Carbohydrate (g)	365	339, 387	359	333, 372	.169
Protein (g)	64.2	57.7, 70.0	66.0	61.0, 70.0	.711
Fat (g)	35.0	30.0, 45.0	36.0	33.0, 42.0	.574
Vitamin A (μg)	316	286, 346	333	284, 352	.301
Vitamin B1 (mg)	0.68	0.64, 0.71	0.67	0.62, 0.7	.431
Vitamin B2 (mg)	0.55	0.52, 0.58	0.54	0.53, 0.55	.467
Vitamin C (mg)	27.6	24.6, 28.4	27.0	24, 27.0	.063
Calcium (mg)	490	437,567	462	449, 529	.997
Magnesium (mg)	45.1	35.2, 334	43.5	34.4, 249	.231
Iron (mg)	8.9	7.9, 9.3	9.7	8.6, 9.7	.020*

Abbreviations: MNSS, male nonsport science; MSS, male sport science; Q1, first quartile; Q3, third quartile.

* Statistically significant difference between male sport science and nonsport science students using Mann–Whitney U‐test: *p* ≤ .005.

**TABLE 3:** Estimated daily median intakes (Q1, Q3) of energy and other selected nutrients of female sport science and nonsport science students in the first‐generation universities of Southern Ethiopia, January 2018.

Variables	Female sport science and nonsport science (*N* = 74)				
	FSS (*n* = 31)		FNSS (*n* = 43)		*p*‐Value
	Median	Quartile (Q1, Q3)	Median	Quartile (Q1, Q3)	
Energy (kcal)	2088	2018, 2146	2027	1919, 2027	.005*
Carbohydrate (g)	374	356, 403.	362	344, 371	.005*
Protein (g)	66.1	63.3, 71.7	66.1	60.0, 70.0	.252
Fat (g)	36.6	32.2, 45.0	35.2	31.6, 41.4	.443
Vitamin A (μg)	321	310, 329	328	292, 354	.220
Vitamin B1 (mg)	0.70	0.66, 0.74	0.67	0.63, 0.70	.018*
Vitamin B2 (mg)	0.55	0.52, 0.58	0.54	0.53, 0.55	.123
Vitamin C (mg)	27.7	25.4, 29.0	27.0	24.0, 28.0	.110
Calcium (mg)	464	434, 553	458	443, 518	.908
Magnesium (mg)	55.4	38.1, 335	44.6	36.4, 249	.060
Iron (mg)	9.0	8.39, 9.62	9.4	8.56, 9.74	.084

Abbreviations: FNSS, female nonsport science; FSS, female sport science; Q1, first quartile; Q3, third quartile.

* Statistically significantly different between groups and declared at U‐test using the Mann–Whitney U‐test: *p* ≤ .05.

Likewise, the average 7 days' estimated daily median intakes of energy, carbohydrate, and vitamin B_1_ were significantly higher in the female sport science group compared to the group from the nonsport science students. However, the median intakes of fat, protein, and other micronutrients such as vitamin A, vitamin B_2_, vitamin C, calcium, magnesium, and iron were not statistically different between the two groups (Table 3).

### 3.3 Prevalence of inadequate intakes of energy and other selected nutrients

More than 50% of the male and female students who participated in this study had inadequate intakes of energy, protein, fat, vitamin A, vitamin B_1_, vitamin B_2_, and magnesium and energy, fat, vitamin A, vitamin B_2_, and magnesium, respectively. Additionally, the prevalence of inadequate intake of protein in female sport science students was more than 50%. However, the prevalence of inadequate intake of carbohydrate, vitamin C, calcium, and iron in both study groups, and additionally the prevalence of inadequate intakes of vitamin B_1_ and protein among the female sport science and nonsport sciences, respectively, were less than 50% (Table 4).

**TABLE 4:** Prevalence of inadequate intakes of energy and other selected nutrients of male and female sport science and nonsport science students in the first‐generation universities of SNNPRS, Ethiopia, January 2018.

Variables	MSS and MNSS (*N* = 92)				FSS and FNSS (*N* = 74)			
	MSS (*n* = 45)	MNSS (*n* = 47)	*p*‐Value	RNI	FSS (*n* = 31)	FNSS (*n* = 43)	*p*‐Value	RNI
	Inadequate intake *N* (%)	Inadequate intake *N* (%)			Inadequate intake *N* (%)	Inadequate intake *N* (%)		
Energy (kcal)	33 (73.3)	30 (63.8)	.327	2650	25 (80.6)	24 (55.8)	.026*	2300
Carbohydrate (gm)	6 (12.8)	6 (12.8)	.936	130	1 (3.22)	3 (7)	.043*	130
Protein (gm)	26 (57.8)	24 (51.1)	.518	56	16 (51.6)	21 (48.8)	.814	46
Vitamin A (μg)	43 (95.6)	41 (87.2)	.157	600	26 (83.9)	29 (67.4)	.110	500
Vitamin B1 (mg)	36 (80.0)	47 (100)	.001*	1.2	4 (12.9)	17 (39.5)	.012*	1.1
Vitamin B2 (mg)	40 (88.9)	44 (93.6)	.421	1.3	28 (90.3)	39 (90.7)	.957	1.1
Vitamin C (mg)	4 (8.9)	5 (10.6)	.778	45	5 (16.1)	10 (23.3)	.452	45
Calcium (mg)	5 (11.9)	6 (12.8)	.807	1000	7 (22.9)	4 (9.3)	.113	1000
Magnesium (mg)	32 (71.1)	32 (68.1)	.753	260	18 (58.1)	27 (62.8)	.681	220
Iron (mg)	11 (24.4)	3 (6.4)	.016*	14	7 (22.6)	7 (16.3)	.495	29

*Note*: Categorical data are presented as a proportion and compared using chi‐square, the cut‐off point of inadequate intake of each nutrient was declared at <2/3 RNI. NB: The inadequacy of for the energy, carbohydrate, protein and selected micronutrients intake from diet was declared considering sex, and the age range of 19–24 years old and below two‐third of the recommended nutrient intake (RNI) for the specific nutrients and energy. For the zinc and iron, the RNI used for this study, were considered the low bioavailability and 10% bioavailability, respectively (FAO/WHO, 2001). The inadequate intake of fat could not be calculated because there is no defined RNI for it. The RNI for energy used in this study considered the age of 18–29.9 years, moderate physical activity level (PAL = 1.75), average weight of the participants (~55 kg), and sex (FAO/WHO/UNU, 2001). The RNI for 19–24 years old was energy (2650 kcal (male), 2300 kcal (female)), carbohydrate (130 g), protein (56 g (male), 46 g (female)), calcium (1000 mg), iron (14 g (male), 29 g (female)), zinc (14 mg (male), 9.8 mg (female)), vitamin B1 (1.2 mg (male),1.1 mg (female)), vitamin B2 (1.3 mg (male), 1.1 mg (female)), vitamin A (600 μg (male), 500 μg (female)), vitamin C (45 mg) (FAO/WHO, 2001; FAO/WHO/UNU, 2001; Institute of Medicine, 2005).

Abbreviations: FNSS, female nonsport science; FSS, female sport science; MNSS, male nonsport science; MSS, male sport science.

* Statistically significant difference between groups and statistically significant difference was declared at *t* ≤ .05.

The prevalence of inadequate intake of vitamin B_1_ and iron was significantly different (***p*** < .05) between the male sport science and nonsport science students, whereas the prevalence of inadequate intakes of energy, carbohydrate, and vitamin B_1_ was significantly different (***p*** < .05) between female sport science and nonsport science student groups. However, the prevalence of inadequacy for the rest of macro‐ and micronutrient intake within the male and female groups (sport science and nonsport science) was not statistically significant (***p*** > .05) (Table 4).

## 4 DISCUSSION

The present study assessed and compared the dietary energy intake and selected nutrient intakes, and compared the prevalence of inadequate intakes of energy and selected nutrients between the students from the sport science and nonsport science groups.

### 4.1 Average dietary energy and nutrient intakes among sport and nonsport students

Our present study revealed that the median intake of iron was between 8.9–9.7 mg for male students and 9.0–9.4 mg for female students. However, these results were lower than the median iron intake by the women of reproductive age from the Butajira district of the Southern Nations, Nationalities, and Peoples' Region (SNNPRS), and Hulla and Shebedino districts of the Sidama region (Bosha et al., 2019). These differences might be due to the consumption of iron‐rich vegetables such as Ethiopian kale in Southern Ethiopia, which includes Sidama and SNNPR regions (Asayehu et al., 2017; Bosha et al., 2019). Likewise, the median iron intake (38.9–40.4 mg) of lactating mothers in the Tigray region was quite higher than reported for female students in the present study (Desalegn et al., 2022). This could be because the staple foods in the northern part of Ethiopia are mainly cereals, known for its iron content. Furthermore, the average dietary iron intake for the university students from Brazil was also higher than what was found in the present study in both sexes (Hartmann et al., 2021).

In this study, the median intake of iron was relatively lower in the male and female students attending sport science department compared to those students in the corresponding nonsport science department group (Tables 2 and 3). These differences might be due to the tendency to incline on preferring more energy‐rich foods such as pasta and bread, and relatively less injera which has high iron, and the former explanation is also supported by the higher intake of energy in the sport science compared to the nonsport science group (Table 3).

The median intake of protein by male and female students was between 64.2–66 mg and 66.1 mg per day, which was higher than found for first‐year female students in Southern Africa, which was 57 mg per day (Verwey et al., 2021). This variation might be due to the number of meals that contain protein‐rich food being small in the latter, unlike that of the sauce usually prepared from lentils or peas are staples in Ethiopian universities. However, the median intake of vitamin A was between 316–333 μg for males and 321–328 μg for female students (Tables 2 and 3), which was lower than that found in the study conducted in Pakistan, in which the average intake of vitamin A by the male and female university students was 703 μg and 605 μg, respectively (Khattak et al., 2002). These could be explained by the difference in the dietary pattern between the countries, for instance, vegetables are among the staples in the Pakistani diet. Moreover, the average vitamin A intake by the students in this study was lower than that found for female adolescents, reproductive‐age women, and men elsewhere in southern Ethiopia (Bosha et al., 2019; Ethiopian Public Health Institute (EPHI), 2013; Yilma et al., 2021).

Furthermore, the median intake of protein and vitamin A by the male and female students from the nonsport science groups was higher than their corresponding male and female students in the sport science group (Tables 2 and 3), which could be due to the pea sauce, and the vegetable sauce which contain carrot, green pepper, and palm oil, which are a rich source of protein and vitamin A, respectively, were preferred and eaten better in amount by the nonsport science student groups (Abebe et al., 2007; Umeta et al., 2005). Furthermore, a study conducted using a National Health and Nutrition Examination Survey 2011–2016 data revealed that the dietary intake of protein (72.7–85.4 g vs. 66.1 g) and vitamin A (581–643 μg vs. 321–328 μg) by adults greater than 18 years old was far higher than we had found in our present study (Fulgoni Iii & Agarwal, 2021), whereas the protein intake by Bangladesh lactating women was consistent with our findings, in which the median protein intake was 67.84 g (Islam et al., 2023).

Furthermore, the median intake of calcium by female students was between 458 and 464 mg, which was far lower than the intake by pregnant women in Italy and the adults in USA (Quattrini et al., 2021; Shah et al., 2021). This big variation could be due to high adherence to the Mediterranean diet, which is known for a high level of dietary calcium and also the larger portion of the size and increased number of meals consumed by pregnant women, as they need extra energy and nutrients demand for pregnant women. The latter could be due to the consumption of milk, which is one of the dietary calcium, was higher.

The median intake of vitamin B_1_ and B_2_ by the male and female students was between 0.67–0.68 mg, 0.67–0.7 mg, and 0.54–0.55 mg (Tables 2 and 3). These intakes were lower than the average intake of vitamin B_1_ (Male: 0.94 mg; Female: 0.83 mg) and vitamin B_2_ (Male: 1.04 mg; Female: 1 mg) by university students in Pakistan (Khattak et al., 2002). Similarly, the median intake of vitamin C (59 mg), calcium (502 mg), and magnesium (218 mg) by the South African university students was higher than the median intake of the same nutrients in the present study, which were between 27–27.6 mg, 458–490 mg, and 43.5–55.4 mg, respectively (Verwey et al., 2021). These differences could be the difference in the dietary practices in general. For instance, the consumption of oats and legumes such as soybean, peanut kidney beans, and oily seeds which are rich sources of vitamin B_1_ and B_2_ could be higher in the Pakistan context, which is not part of the components of the recipes in the selected universities in Ethiopia.

The median intake of vitamin C by the female students was lower than the intake of the same nutrient by the women at reproductive age in the Butajira district, which could be due to the consumption of vitamin C‐rich Ethiopian kale and green pepper was better in the latter group (Asayehu et al., 2017). Furthermore, the intake of calcium and magnesium was also less by the students in the present study compared to that was found for women from the Sidama region, which might be the consumption of Enset (*Ensete ventricosum*) and diversified cereals and legumes in the rural areas of Sidama region (Bosha et al., 2019).

### 4.2 Inadequate intakes of energy and nutrients among sport and nonsport students

In the present study, the prevalence of inadequate intake of vitamin B_1_ was significantly (*p* < .05) higher in students from nonsport science department group compared to their counter sport science group in the same sex (Table 4). However, vitamin B_1_ intake by most male students was inadequate compared to the female students regardless of the department they were studying (80–100% vs. 12.9%–39.5%, respectively). The former could be related to the preference and consumption of cereals and legume‐based foods by the sport science department students, as there is a perception that energy‐rich foods are more important for athletes and those who are engaged in more vigorous exercise. However, the latter might be due to the concentration of vitamin B_1_ being low even in those known as vitamin B_1_‐rich foods and the food provided both to male and female students are the same even if the requirement is higher for males in the same age group (FAO/WHO, 2001).

The prevalence of inadequate intake of dietary iron was 6.4%–24.4% for the male student group and 16.3%–22.6% for the female student group (Table 4). The latter results were lower than those reported for females in late adolescent age in the Wolaita district in SNNPRS, which was 53%, and for women at reproductive age in the Sidama region (32.1%–43.7%) (Bosha et al., 2019; Yilma et al., 2021). These variations might occur because the estimated intake of iron in the study population had a wider range and skewed to the left, which might be explained by the difference in the socioeconomic status which determines the quality of diet consumed in community settings in Ethiopia, unlike small variation in the intake by university students, due to the same food provided in the cafeteria. Yet, the prevalence of inadequate intake of iron was significantly (*p* < .05) higher in the male students from sport science group compared to the male nonsport science (Table 4). The reason explained for the lower median intake of iron by the sport science students also works here. Yet, besides increasing the quantity of iron intake in the diet, improving the bioavailability of iron, by increasing the consumption of animal source food, which is the source of iron, and also the consumption of vitamin C‐rich source foods, should be given attention in the universities. Even if the problem of inadequate intake of vitamin C was also important with that of iron, there was no significant difference between the students from the sport science and nonsport science departments (Table 4). Moreover, as the production of iron‐rich plant‐source food is higher, the intake of dietary iron is high in Ethiopia despite the bioavailability being much lower (Guja et al., 2023), which is one of the main problems for the high burden of iron deficiency anemia unless the consumption of bioavailable animal source food consumption is improved (Central Statistical Agency (CSA), 2017).

Similarly, the prevalence of inadequate intake of calcium was between 11.9%–12.8% and 9.3%–22.9% for male and female students, respectively, and this result was not significantly different between sport science and nonsport science groups in both sexes (Table 4). However, almost all female adolescents and women of reproductive age from southern Ethiopia had taken dietary calcium below the daily average requirement (Asayehu et al., 2017; Bosha et al., 2019). Another study on 6‐ to 23‐month‐old children in the same region also revealed that the prevalence of inadequate intake of calcium was high (76.8%) (Feyisa et al., 2020). The variation could be the differences in the study settings, which was institutional and the study participants in the present study consumed their food from the university cafeteria, unlike the latter, in which the consumption practice could be dependent on multiple factors that can affect the consumption practices in rural households that are majorly dependent on what they are producing in their farm.

In this study, the prevalence of inadequate intake of energy was between 55.8%–80.6% and 63.8%–73.3% for female and male students groups, respectively. This could be related to the quantity/portion size of the food provided to the students in the selected university cafeteria being small in general or the students were consuming might be less which is associated with the quality of the food. A comparable proportion (61.4%–64.4%) of inadequate energy intake was reported for women of reproductive age in the Sidama region (Bosha et al., 2019). However, the inadequate intake of energy was significantly (*p* < .05) higher in the female students from sport science compared to the same sex in the nonsport science group. This might be related to the foods prepared from low fat are consumed by the female sport science group compared to their counter male group. This was also clearly observed by the higher inadequate intake of fat in the female sport science group and the decrease in the portion size of the foods which covered the majority of the meal provided at the student cafeteria, which is injera and bread (Table 4). This could be associated with food preference or choice. In addition to this, low fat intake is associated with poor fat‐soluble vitamin absorption. Therefore, taking the proper amount of fat is recommended. Similarly, protein is a component of all our cells, supporting muscle growth, immune and circulatory health, tissue structure, endocrine function, and enzymatic reactions. In the present study, a slightly higher proportion of male students had inadequate intake of protein (51.1%–57.8% vs. 48.8%–51.6%, respectively); however, there was no significant difference between the male and also female students attending sport science and nonsport science departments (Table 4). Therefore, improving the energy intake should consider increasing the diversity of protein source foods and also the intake of fat considering the recommended contribution proportion of carbohydrate, protein, and fat for energy requirement. Unlike our present finding which reported 48.8%–51.6% of inadequate intake of protein, 86% of adult women of the Caribbean island of Barbados who were at high burden of diabetes and cardiovascular disease had fulfilled the recommended daily allowance (RDA) of dietary protein based on two 24‐h dietary recall data (Harris et al., 2020). This difference might be related to the good dietary practices associated with dietary recommendations and adherence to manage the risk of the diseases for later cases.

In this study, the prevalence of inadequate intake of vitamin A from the diet was not significantly different between the sport science and nonsport science student groups in both sexes, but it was between 87.2%–95.6% and 67.4%–83.9% for male and female student groups, respectively. A lower prevalence of inadequate intake of vitamin A was reported for the women of reproductive age in Butajira district (0%), Sidama region (23.7%–44.2%), and for the SNNPR region (41.3%) (Asayehu et al., 2017; Bosha et al., 2019; Ethiopian Public Health Institute (EPHI), 2013). This discrepancy could be due to the consumption of vitamin A‐rich vegetables and fruits being better in southern Ethiopia, which is not usually the case in the university students' diet.

The prevalence of inadequate intake of dietary vitamin B_2_ and magnesium was not significantly different between the sport and nonsport science groups. However, nine out of the 10 (88.9%–93.6%) and about one‐third (62.8%–71.1%) of the university students did not meet two‐thirds of the RNI for vitamin B_1_ and magnesium, respectively. Considering the high prevalence of inadequate intake of energy and majority of nutrients and they were not significantly different between the students from sport science and nonsport science groups in both sexes, the quality of diet provided to the selected university students in southern Ethiopia was poor and not satisfying the nutrient requirements. Moreover, the problem could be more serious for the students attending sport science department. Therefore, considering the energy and nutrient demand based on their sex, age, and physical activity level, efforts to improve the dietary energy and nutrient intakes of sport science and the whole university students taking cafeteria services should include by revision of menu and meal plans.

The suboptimal dietary energy and nutrient intake among university students in general and specifically in those extra energy and nutrient demand disciplines as part of the learning process like in sport science disciplines should catch the attention of public universities and the government to give due to improvement of the quality of diets provided in university cafeterias. This can be done majorly through continuous follow‐up on the provided meal if satisfying the recommended energy and nutrient intake of students, preparing the meal plan based on the daily dietary recommendation, and revision of the food budget allocated, as the food price is ever‐changing dramatically in the country and globally. This should not undermine the necessity of distributing courses that demand rigorous physical exercise as part of academic requirements along different semesters of the academic years by revising sport science and other curriculum to minimize the imbalance between the energy and nutrient expenditure and the demand to maintain the health of university students.

#### 4.2.1 Strengths and limitations of the study

Even if this study has some limitations, which should be considered during the interpretation of the results, much strength has been also noted. The limitations are: (1) the cross‐sectional nature of the study could not support us to catch the variability in the meal prepared and provided, which is usually affected by seasonality and also cost; (2) the study only included the first‐generation universities resided in southern Ethiopia, which may not represent the whole universities in the country; (3) the dietary assessment method is usually prone to systematic error and also estimation, which may not tell the actual intake, in which errors may emanate from the survey instruments, estimation, and food composition databases; and (4) the Amharic version questionnaire was not checked for its reproducibility and reliability. The strengths are: (1) the dietary assessment method which was used in this study is food weight recording “Gold Standard”; (2) 7‐day dietary intake data were collected, which supports identifying the usual intake of the students eating at the selected university cafeteria during the study semester, and also identifying the inadequate intake of energy and the selected nutrients at the individual student level; and (3) the study also included both sexes and the students from sport science departments, known with more than 50% of the courses in the curriculum require high physical activity and other nonsport science students for comparison.

## 5 CONCLUSION

The average intake of iron by the male sport science students was higher than the male nonsport science students. Similarly, female sport science students were shown to take higher dietary energy, carbohydrate, and vitamin B_1_ intakes compared to female nonsport science students. Furthermore, the prevalence of inadequate intake of vitamin B_1_ for the students from the nonsport science department was significantly higher than the students from the sport science group, unlike for the carbohydrate, for which the proportion of female students with inadequate intake was significantly higher than in the female attending the nonsport science department in the selected first‐generation universities located in southern Ethiopia. Moreover, the prevalence of inadequate intake of energy, protein, fat, vitamin A, B_2_, and magnesium was very high (>50%) in the students' population regardless of the department they were attending and their sex difference, except that of the inadequate intake of vitamin B_1_ in the male students' population.

In general, the dietary nutrient intakes of the students in the selected universities are suboptimal; however, the problem is more serious in the students attending sport science department. Activities that will improve the dietary intake of the selected university students should include weakly meal plan revision considering the average recommended nutrient intake of the students, healthy diet, and the budget of the students. Special emphasis should be given to sport science students with more energy expenditure related to their usual vigorous physical activity.

A further longitudinal study should be conducted considering foods consumed outside the cafeteria during different semesters, and by other faculty and different university students to understand the dietary intake of sport science, nonsport science students, and the whole university students in general.

## AUTHOR CONTRIBUTIONS

**Kebede Awgechew:** Conceptualization (equal); data curation (equal); formal analysis (equal); funding acquisition (equal); investigation (equal); methodology (equal); project administration (equal); resources (equal); supervision (equal); visualization (equal); writing – original draft (equal); writing – review and editing (equal). **Beruk Berhanu Desalegn:** Conceptualization (equal); data curation (equal); formal analysis (equal); investigation (equal); methodology (lead); software (lead); supervision (equal); writing – original draft (equal); writing – review and editing (equal). **Addisalem Mesfin:** Conceptualization (equal); data curation (equal); formal analysis (equal); funding acquisition (equal); investigation (equal); methodology (equal); project administration (equal); resources (equal); supervision (equal); writing – original draft (equal).

## FUNDING INFORMATION

This research project was funded by Hawassa University (Disciplinary Research for 2017).

## CONFLICT OF INTEREST STATEMENT

The authors declare that they have no competing interests.

## ETHICAL APPROVAL

Ethical clearance was obtained from the Institutional Review Board (IRB) of Hawassa University's College of Medicine and Health Sciences. Permission was also obtained from Hawassa, Dilla, and Arba Minch universities using the support letter written and provided by the School of Nutrition, Food Science and Technology, Hawassa University. Additional permission was also obtained from the study participants before the first survey day during the waiting time for the cafeteria service. During the first contact, the purpose of the study and the confidentiality of the data to be collected were explained to each participant and ensured using an individual three‐digit code. At the same time, the participants were also told that their participation in this study was based on their willingness and whenever they felt to discontinue from the participation during the data collection, withdrawal from the study was possible.

## CONSENT FOR PUBLICATION

All authors agreed to publish the manuscript.

## Supporting information


Table S1

## Data Availability

The datasets analyzed during the current study are available from the corresponding author upon reasonable request.
