# Supplementary material for: Inadequate intake of energy and nutrients: A comparative cross‐sectional study between sport and nonsport science university students of southern Ethiopia
Source: Food Sci Nutr. 2023 Nov 2;12(1):590–601. doi: 10.1002/fsn3.3779 (PMC10804097; doi:10.1002/fsn3.3779)
Supplement: Supplementary file 1 — Table S1 [file FSN3-12-590-s001.docx]

Supplementary 1: Sample size calculation for the study

| **Input** | **Output** | **Remark** |
| --- | --- | --- |
| Tail(s)=Two | Noncentrality parameter δ=3.278719 | By using G-power software version 3.0 |
| Effect size (Cohen’s d=0.5) | Critical t=1.974017 |  |
| α err probability=0.05 | Df=170 |  |
| Power (1-β err probability)=0.90 | Sample size group 1=**90** |  |
| Allocation ratio N2/N1= 1.1 | Sample size group 2=**82** |  |
|  | Total sample size=**172** |  |
|  | Actual power=0.903230 |  |

DF = degree of freedom.
